# Local ancestry and selection in admixed Sanjiang cattle

**DOI:** 10.1007/s44154-023-00101-5

**Published:** 2023-08-03

**Authors:** Yang Lyu, Yaxuan Ren, Kaixing Qu, Suolang Quji, Basang Zhuzha, Chuzhao Lei, Ningbo Chen

**Affiliations:** 1 Key Laboratory of Animal Genetics, Breeding and Reproduction of Shaanxi Province, College of Animal Science and Technology, Northwest A&F University, Yangling, China; 2 Academy of Science and Technology, Chuxiong Normal University, Chuxiong, China; 3 Institute of Animal Husbandry and Veterinary Science, Tibet Academy of Agricultural and Animal Husbandry Sciences, Lhasa, China

**Keywords:** Whole-genome sequencing, Local ancestry, Selection, Adaptation

## Abstract

The majority of native cattle are *taurine* × *indicine* cattle of diverse phenotypes in the central region of China. Sanjiang cattle, a typical breed in the central region, play a central role in human livelihood and have good adaptability, including resistance to dampness, heat, roughage, and disease, and are thus regarded as an important genetic resource. However, the genetic history of the successful breed remains unknown. Here, we sequenced 10 Sanjiang cattle genomes and compared them to the 70 genomes of 5 representative populations worldwide. We characterized the genomic diversity and breed formation process of Sanjiang cattle and found that Sanjiang cattle have a mixed ancestry of indicine (55.6%) and taurine (33.2%) dating to approximately 30 generations ago, which has shaped the genome of Sanjiang cattle. Through ancestral fragment inference, selective sweep and transcriptomic analysis, we identified several genes linked to lipid metabolism, immune regulation, and stress reactions across the mosaic genome of Sanjiang cattle showing an excess of taurine or indicine ancestry. Taurine ancestry might contribute to meat quality, and indicine ancestry is more conducive to adaptation to hot climate conditions, making Sanjiang cattle a valuable genetic resource for the central region of China. Our results will help us understand the evolutionary history and ancestry components of Sanjiang cattle, which will provide a reference for resource conservation and selective breeding of Chinese native cattle.

**Supplementary Information:**

The online version contains supplementary material available at 10.1007/s44154-023-00101-5.

## Introduction

Domesticated cattle are the main livestock acting as a driving force in agriculture and transportation, playing a significant role in agricultural society. Domestic cattle are mainly divided into humpless taurine cattle (*Bos taurus taurus*) and humped indicine cattle (*Bos taurus indicus*) (Decker et al. 2014). The geographical distribution pattern of domestic cattle in the world is very regular and closely related to the climate background. Taurine cattle can adapt to temperate and cold climates, mainly in the Northern Hemisphere (Buggiotti et al. 2021; Xia et al. 2023). Indicine cattle are heat-resistant and drought-resistant and adapted to tropical and subtropical climates. They are mainly distributed in the equatorial region and the Southern Hemisphere, mostly in South Asia, Southeast Asia, southern East Asia, and Africa (Utsunomiya et al. 2019; Zhang et al. 2020).

China is rich in bovine species resources, and there are 55 native cattle breeds with distinct phenotypes (MacHugh et al. 1997). Native cattle in the central region of China, also called yellow cattle, have undergone long-term artificial selection and natural selection, often leaving unique regions in the genome, which are considered important genetic resources due to their distinct characteristics. Recently, native Chinese cattle have been shown to have three types of ancestry: Eurasian taurine and East Asian taurine in northern China and Chinese indicine in southern China (Chen et al. 2018), which are inseparable from the complex genetic background and domestication history of Chinese cattle. Following their contact, a north-to-south taurine-to-indicine cline of cattle was established. Intermediate taurine-indicine breeds in the central region of China exhibit different combinations of taurine and indicine ancestries at both phenotypic and genomic levels, which serve as a major labor force in agricultural production and are well known for their endurance and adaptation. Different taurine × indicine admixture proportions increase diversity and provide new genetic resources for human and natural selection. However, the history formation processes of different hybrid breeds in the central region of China warrant further investigation.

**Fig. 1:**
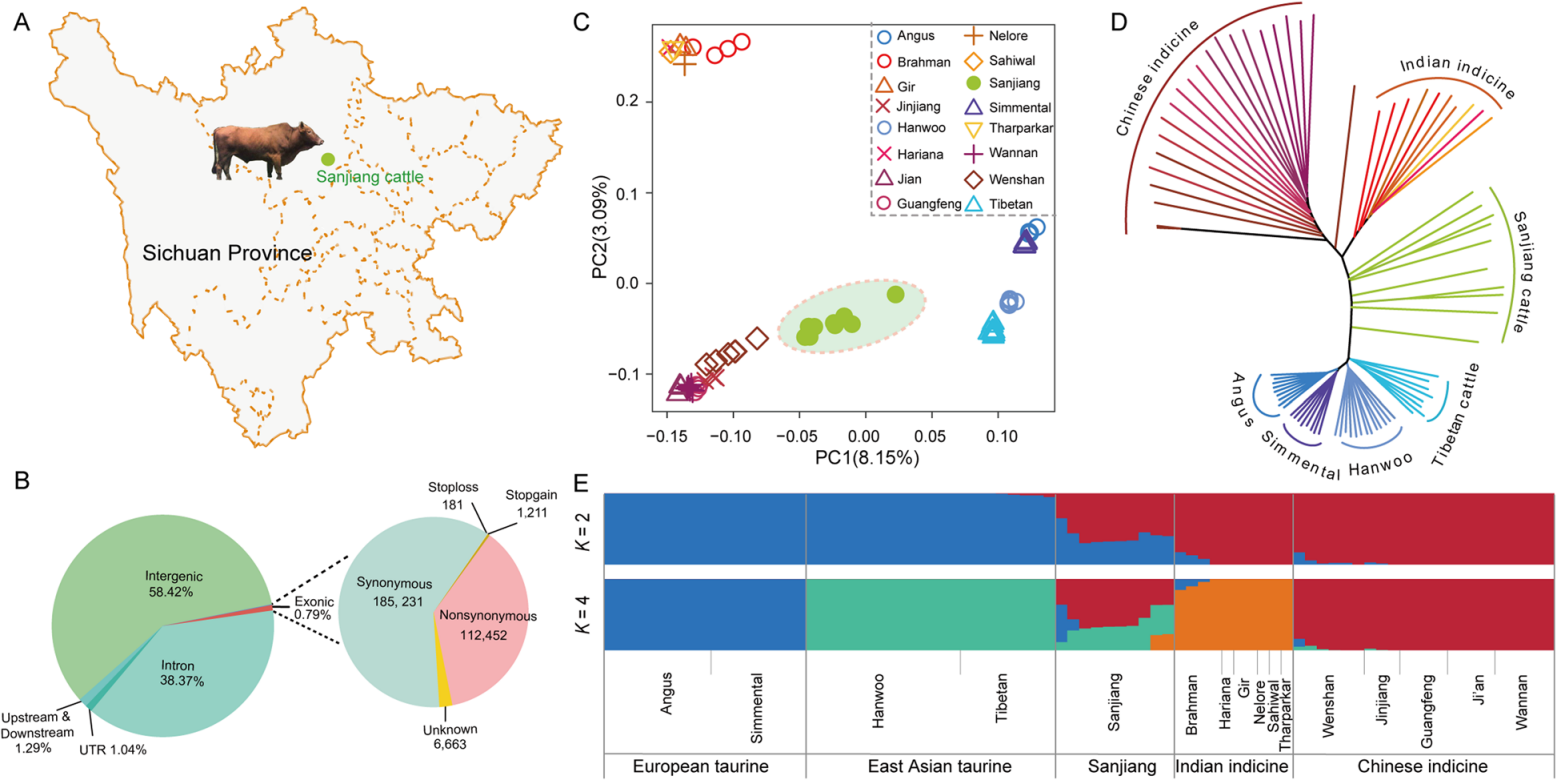
Population structure and relationships of Sanjiang cattle in comparison to several possible ancestral breeds. **A** The distribution map of the Sanjiang cattle. **B** Functional classification of the detected SNPs. **C** Principal component analysis. **D** Neighbor-joining tree of the relationships between Sanjiang cattle and possible ancestors. **E**. Model-based clustering of cattle breeds using ADMIXTURE with *K* = 2 and *K* = 4. Breeds are colored according to their geographic region and labeled with breed names

Sanjiang cattle, distributed in Wenchuan County, Sichuan Province (Fig. 1A), is one of the local well-known cattle breeds (Fig. S1) and a typical native cattle breed in the hybrid region of China. This cattle breed has a relatively large body size and displays a long service life, good adaptability, low disease susceptibility and high endurance under unfavorable feeding conditions. It is urgent for animal husbandry workers to study the genetic diversity and protect resources of Sanjiang cattle. However, the origin and genomic background of Sanjiang cattle, the timing of taurine × indicine admixture event(s) and their impacts on the economic traits and adaptation of local cattle remain unknown. With the development of sequencing technology and the reduction of sequencing costs, whole genome sequencing (WGS) technology has been responsible for several milestones in understanding the origin and evolution of cattle and identifying their population structure and genomic regions associated with important economic and environmental adaptation traits (Lee et al. 2013; Tsuda et al. 2013; Xia et al. 2021). Downstream WGS analysis and differentially expressed genes can provide a basis for designing genetic breeding strategies to improve the adaptability and productivity of cattle.

In the present study, we identified the genome diversity, population structure, and global and local ancestry proportions of Sanjiang cattle. We date a main taurine × indicine admixture event and assess the present genome ancestry of Sanjiang cattle and present selected regions with excessive segments showing an excess of taurine or indicine ancestry in Sanjiang cattle. Moreover, we combined transcriptome data to further confirm the reliability of the putatively selected genes. These data provide valuable genomic resources for promoting molecular breeding and genetic improvement of Sanjiang cattle and support that a combination of these two ancestries is at the root of the success of breed formation in the central region of China.

## Results

### Sequencing and identification of single nucleotide polymorphism (SNP)

Individual genomes of 10 Sanjiang cattle were jointly genotyped with publicly available genomes for genetic background analysis. A total of 70 cattle genomes were added as a control group, including samples of European taurine, East Asian taurine, Indian indicine and Chinese indicine groups. A total of 80 samples were therefore used in this study, with an average sequence coverage of ~ 11.5 × and an average mapping rate of 99.39% (Table S1). A total of ~ 38.7 million biallelic SNPs were annotated through ANNOVAR (Wang et al. 2010) in 80 samples. Most of the SNPs were located in intergenic (58.42%) and intronic regions (38.37%), while the rest were located in the regions upstream and downstream (1.29%) of open reading frames and untranslated regions (1.04%). In addition, exons contained 0.79% of the total SNPs, with 112,452 nonsynonymous SNPs and 185,231 synonymous SNPs (Fig. 1B and Table S2).

### Patterns of genomic variation

The nucleotide diversity analysis showed that indicine origin cattle, including Chinese indicine cattle, Sanjiang cattle and Indian indicine cattle, had significantly higher nucleotide diversity than taurine cattle (Fig. S2). The largest number of SNPs was found in Chinese indicine cattle, followed by Sanjiang cattle and Indian indicine cattle, which showed much greater numbers of SNPs than the taurine groups. Similar results were obtained when considering the numbers of breed-specific SNPs (Fig. S3). In contrast, genome-wide linkage disequilibrium (LD) decreased with increasing physical distance between markers. When the r^2^ value decayed to half of the maximum value, the decay distance in Sanjiang cattle (3.2 kb) was longer than those in Indian indicine (1.5 kb) and Chinese indicine (1.6 kb) cattle but shorter than those in Hanwoo (4.8 kb), Tibetan (9.8 kb), Simmental (10.4 kb), and Angus (11.7 kb) cattle (Fig. S4). The kinship was estimated using the method of inferring IBD fragments in KING software, and there were no individuals with recent genetic relationships (≤ 2nd degree relatives) among the 10 Sanjiang cattle (Table S3). In addition, runs of homozygosity (ROH) analysis was performed on each individual. The results showed that these settings get the expected number (maximum number is 1,925) and total length (maximum length is 774,586 Mb) of ROHs (Fig. S5, Table S4). The Angus and Simmental cattle tended to have more and longer ROHs than Sanjiang cattle (Fig. S5, Table S4), which also reflects the relatively short-term artificial breeding of Sanjiang cattle.

### Population structure and relationships

Principal component analysis (PCA), ADMIXTURE analysis, and neighbor-joining (NJ) tree analysis were performed to explore the genetic relationships among Sanjiang cattle and other cattle groups. The first PC explained 8.15% of the total variation and was driven by variation between *B. taurus* and *B. indicus*. The second PC, explaining 3.09% of the total variation, geographically separated the different indicine and taurine groups. Sanjiang cattle were at an intermediate position between Chinese indicine and East Asian taurine cattle (Fig. 1C). Similar results were observed in the NJ tree and ADMIXTURE analysis (Fig. 1D and E). The cattle breeds were divided into taurine or indicine ancestry at *K* = 2, while at *K* = 3, East Asian taurine cattle were separated from European taurine cattle, and Indian indicine cattle were further separated at *K* = 4. Consequently, Sanjiang cattle showed a proportion of four types of ancestry: Chinese indicine (0.556), East Asian taurine (0.332), European taurine (0.068) and Indian indicine (0.044).

### Local ancestry inference of Sanjiang cattle

**Fig. 2:**
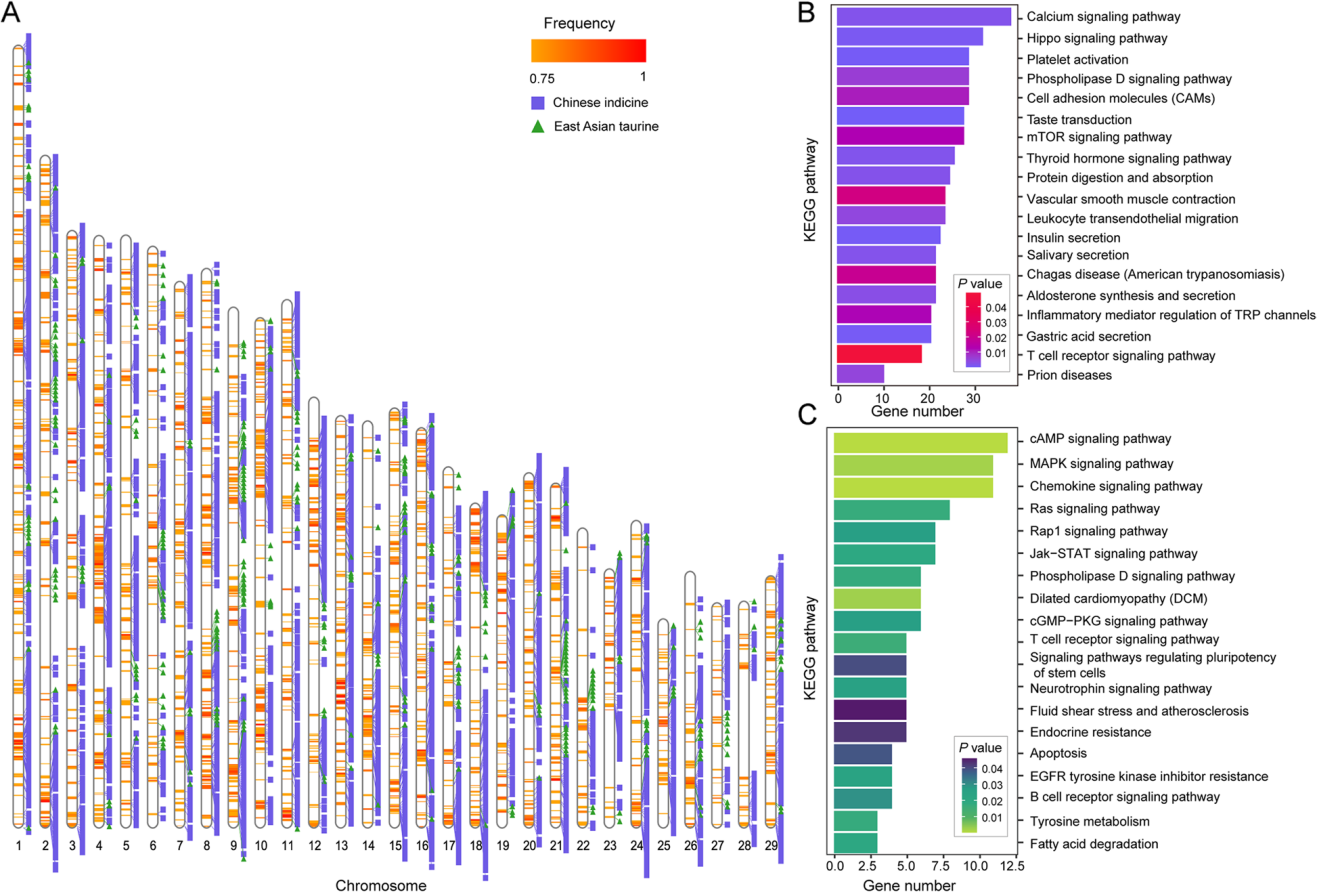
Identification of the local segments in which proportions of a certain ancestry were significantly higher than the proportion in the whole genome in Sanjiang cattle. **A** Distribution of the local segments with proportions of Chinese indicine and East Asian taurine ancestries. **B** The KEGG pathways from the enrichment analysis of genes with excessive Chinese indicine proportions. **C** The KEGG pathways from the enrichment analysis of genes with excessive East Asian taurine proportions

Having established the level of taurine × indicine admixture of Sanjiang cattle, we then estimated the timing of its generation using admixture LD decay. We first employed a single-pulse admixture model using ALDER (Loh et al. 2013). In Sanjiang cattle, the admixture time was dated to 38.27 ± 10.56 generations ago, which supports a recent admixture signal. And we performed fastGLOBETROTTER to determine the admixture dating of Sanjiang cattle, and inferred the mixed signal was about 30.72 generations ago, which was within the date range estimated by ALDER. We then inferred local taurine and indicine ancestries across Sanjiang genomes using LOTER (Dias-Alves et al. 2018). Chinese indicine, East Asian taurine, European taurine, and Indian indicine cattle were selected as reference panels. Their autosomes were expurgated into 30,059 segments. The segments with frequencies of at least 0.75 and lengths of at least 1,000 bp were regarded as high-frequency ancestral fragments by filtering at a *P* value < 0.01. Ultimately, 2,629 Chinese indicine segments, 399 East Asian taurine segments, two European taurine segments and five Indian indicine segments were retained (Fig. 2A and Table S5). The maximum lengths of segments in the Chinese indicine, East Asian taurine, European taurine and Indian indicine groups were 1,191,774 bp, 942,233 bp, 448,738 bp and 123,757 bp, respectively (Table S5). For excessive Chinese indicine segments in Sanjiang cattle, 3,561 genes were annotated (Table S5). These genes were enriched in Kyoto Encyclopedia of Genes and Genomes (KEGG) pathways (*P* value < 0.05) of insulin secretion, inflammatory mediator regulation of TRP channels, platelet activation, T-cell receptor signaling pathway, and thyroid hormone signaling pathway (Fig. 2B and Table S6). For excessive East Asian taurine segments in Sanjiang cattle, 405 genes were annotated (Table S5). The enrichment analysis revealed that these genes were associated with some KEGG pathways (*P* value < 0.05), such as regulation of the MAPK signaling pathway, phospholipase D signaling pathway, fatty acid degradation, and tyrosine metabolism (Fig. 2C and Table S7).

### Selection signatures with an excess of indicine or taurine ancestry in Sanjiang cattle

Sanjiang cattle, considered to have a hybrid origin involving indicine and taurine cattle, haplotypes of indicine or taurine ancestry may confer a relative adaptive advantage under selection pressures. Accordingly, we applied four methods (composite likelihood ratio (CLR), the integrated haplotypes score (*iHS*), *F*_ST_, and *θ*π-ratio) to detect haplotypes related to selection in Sanjiang. The overlapping regions of two or more methods (top 1%) were considered candidate selective regions.

**Fig. 3:**
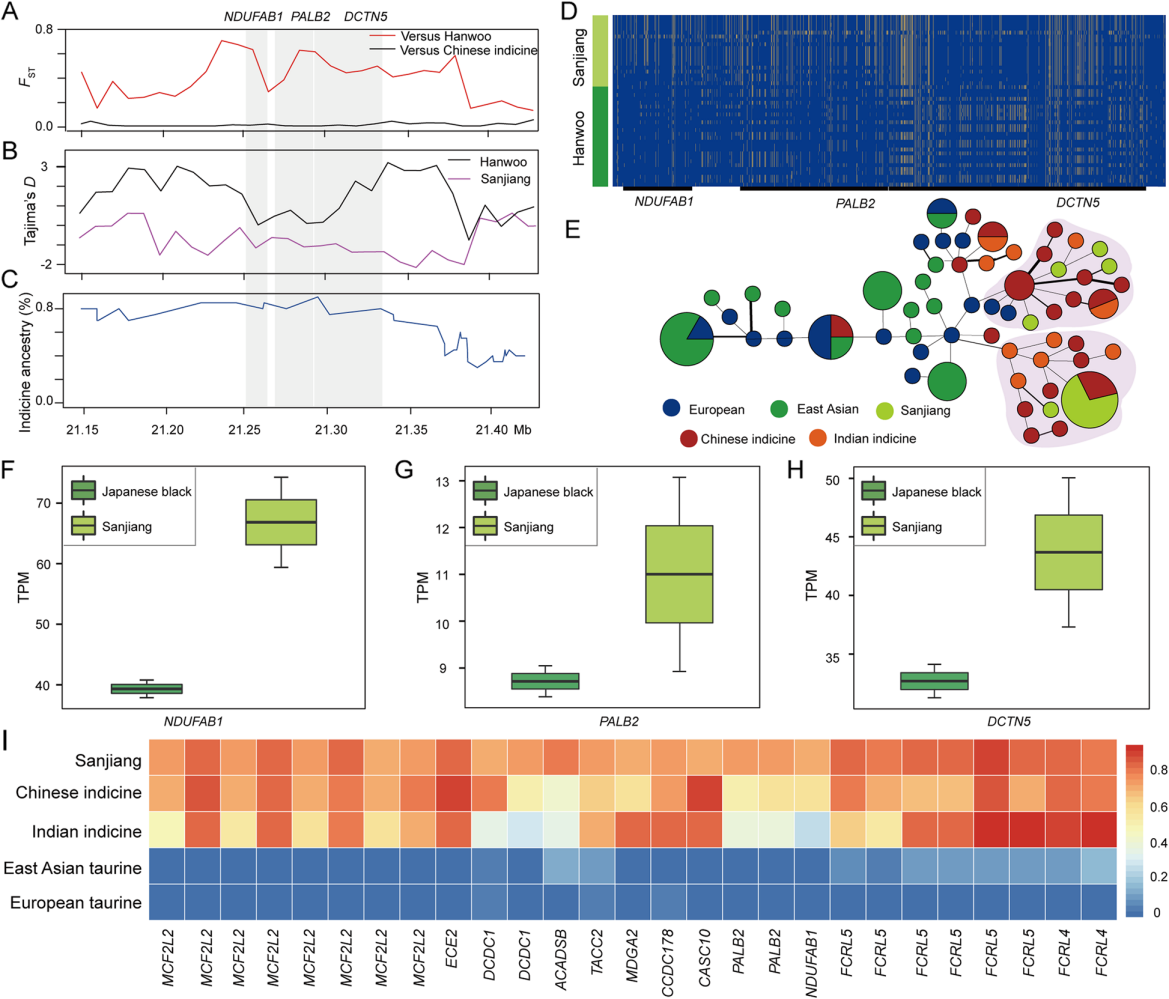
Example of candidate selective loci on *Bos taurus* autosome (BTA) 25 with an excess of indicine ancestry. **A** Pairwise *F*_ST_ values for each 5-kb window with a 2-kb step around the candidate loci (BTA25: 21.15–21.40 Mb). The red line indicates the pairwise *F*_ST_ values between Sanjiang and Hanwoo cattle. The black line indicates the pairwise *F*_ST_ values between Sanjiang and the Chinese indicine cattle. **B** Tajima’s *D* in each nonoverlapping 2-kb window around the candidate loci (BTA25: 21.15–21.40 Mb). **C** Average indicine ancestry (%) around the candidate loci (BTA25: 21.15–21.40 Mb). **D** SNPs were used to construct haplotype patterns. The major allele at each SNP position in Sanjiang cattle is colored orange, and the minor allele is colored blue. **E** The haplotypes of the relationships among Sanjiang cattle, Chinese indicine, Indian indicine, East Asian taurine, and European taurine cattle around the candidate loci (BTA25: 21.15–21.40 Mb). **F**–**H** Comparison of transcripts per million (TPM) in lung tissue of Japanese black cattle and Sanjiang cattle. **I** Twenty-seven missense SNPs with high frequency alleles present in Sanjiang cattle (> 70%) with an excess of indicine ancestry but low frequency of taurine ancestry (MAF < 0.2)

For indicine ancestry, we used East Asian taurine cattle (Hanwoo) as a reference group to detect selection signatures with an excess indicine ancestry in Sanjiang cattle, and a total of 175 candidate regions under selection containing 173 genes were identified (Tables S8, S9, S10 and S11). These candidate genes were significantly overrepresented (*P* value < 0.05) in the cAMP signaling pathway, thyroid hormone signaling pathway, and Rap1 signaling pathway (Table S12). The cAMP signaling pathway plays an important role in neuromodulation and the immune response (Zhou et al. 2019). The thyroid hormone signaling pathway plays a critical role in heat stress and metabolic homeostasis in animals (Maloyan and Horowitz 2022; Matesanz et al. 2017). Moreover, 70 genes were identified in the excessive indicine segments (Table S5). Among them, six genes played a role in the immune response (*PALB2*, *DCTN5*, *FCRL4*, *FCRL5*, *PTGER3*, and *C1QTNF4*), and five genes were related to stress reactions (*EMC4*, *NDUFAB1*, *NOD1*, *GARS*, and *PSMD2*). We found a region on *Bos taurus* autosome (BTA) 25:21.25–21.35 Mb containing three genes (*NDUFAB1*, *PALB2*, and *DCTN5*) that showed a significantly high *F*_ST_ value, low Tajima’s *D*, high indicine ancestry, and long haplotype pattern in Sanjiang cattle (Fig. 3A-D). The haplotypes of the three genes (*NDUFAB1*, *PALB2*, and *DCTN5*) in Sanjiang cattle originated from Chinese indicine (Fig. 3E). Japanese black cattle are classic East Asian cattle. Transcriptome analysis of the lung tissues of Japanese black cattle and Sanjiang cattle could shed light on how the genes retained by the indicien ancestors are expressed in different environments. The results showed that these genes were differentially expressed in the lung tissues of Japanese black cattle and Sanjiang cattle (Fig. 3F-H), further illustrating their contribution to the evolution of Sanjiang cattle. Of these, 27 Sanjiang cattle high-frequency derived alleles were detected (minor allele frequency (MAF) > 0.7), most of which were high frequencies in the indicine cattle but with a low frequency in taurine cattle (MAF < 0.2) (Fig. 3I and Table S13).

**Fig. 4:**
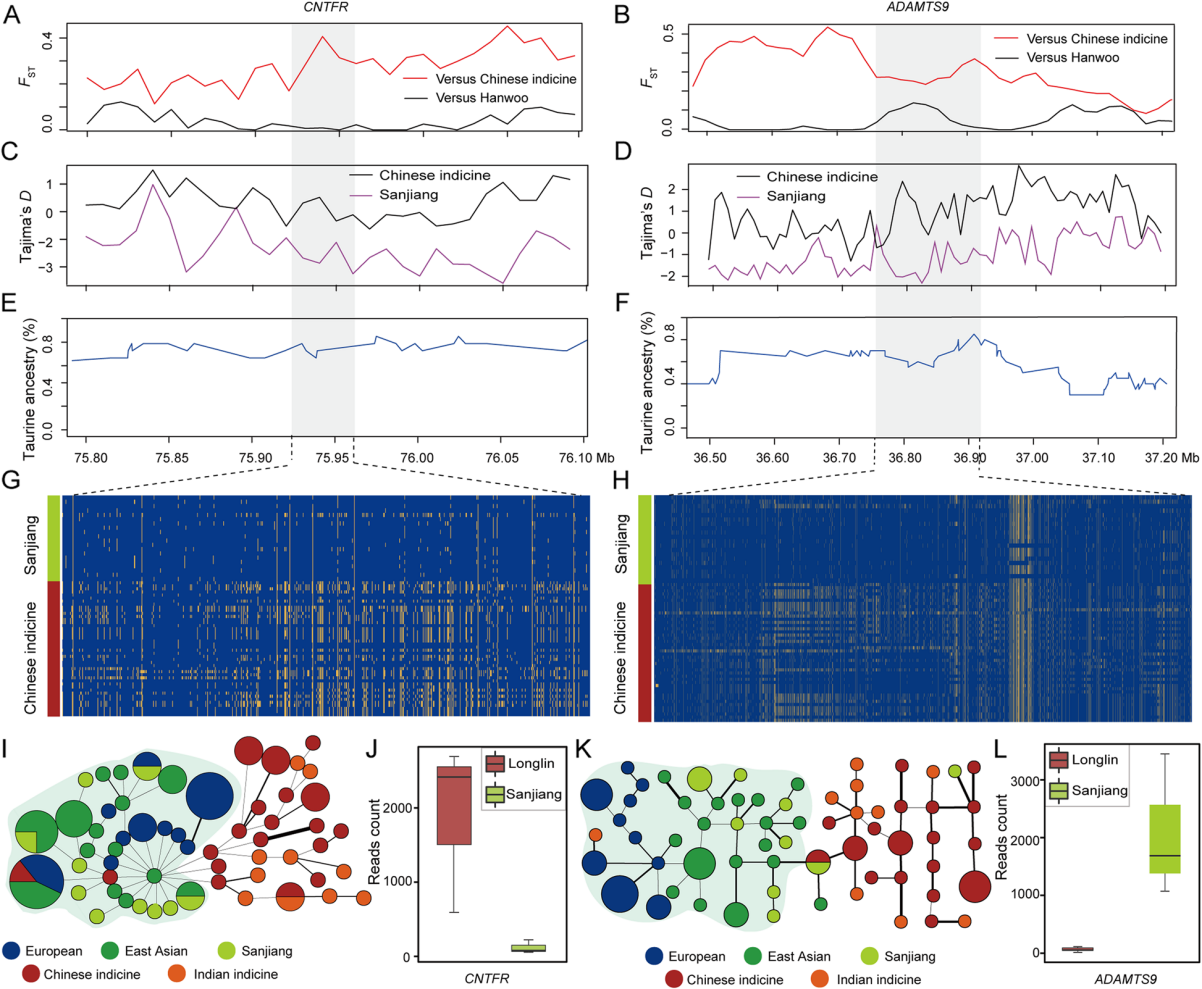
Examples of candidate selective regions with excess taurine ancestry. **A**, **B** Pairwise *F*_ST_ values for each 5-kb window with a 2-kb step around the candidate regions. The red line indicates the pairwise *F*_ST_ values between the Sanjiang and Chinese indicine cattle. The black line indicates the pairwise *F*_ST_ values between Sanjiang and Hanwoo cattle. **C**, **D** Tajima’s *D* in each nonoverlapping 2-kb window around the candidate regions. **E**, **F** Average taurine ancestry (%) around the candidate regions. **G**, **H** SNPs with MAF > 0.05 were used to construct haplotype patterns. The major allele at each SNP position in Sanjiang cattle is colored orange, and the minor allele is colored blue. **I** Haplotypes of the relationships among Sanjiang cattle, East Asian taurine, European taurine, Chinese indicine, and Indian indicine cattle on the *CNTFR* gene. **J** Distribution of reads mapped to the *CNTFR* gene. **K** Haplotypes of the relationships among Sanjiang cattle, East Asian taurine, European taurine, Chinese indicine, and Indian indicine cattle on the *ADAMTS9* gene. **L** Distribution of reads mapped to the *ADAMTS9* gene

For taurine ancestry, we used Chinese indicine cattle as a reference group to detect selection signatures with an excess of taurine ancestry in Sanjiang cattle (Fig. 4A-H). A total of 303 candidate regions under selection containing 324 genes were detected (Tables S8, S9, S14 and S15). These candidate genes were significantly overrepresented (*P* value < 0.05) in fatty acid degradation, the NF-kappa B signaling pathway and the MAPK signaling pathway (Table S16). More specifically, we also found that the 95 genes of 80 regions were retained from the excessive segments of East Asian taurine ancestry (Table S5). Among the excessive segments, many genes associated with shaping particular characteristics of the populations are present within these regions. *CNTFR*, *ADAMTS9*, *SIGMAR1*, *TSEN2, ADRB1, SLC35F3*, *EPRS, CCL21, LPIN3*, and *PPARG* were found to be potentially associated with lipid metabolism and meat quality, and *LEKR1*, *LMBR1*, and *FANCA* were found to be potentially associated with growth traits. For example, we found that the regions of the *CNTFR* and *ADAMTS9* genes in Sanjiang cattle showed a significantly high *F*_ST_ value, low Tajima’s *D* value, and high taurine ancestry (Fig. 4A-F). The haplotypes of the *CNTFR* and *ADAMTS9* genes in Sanjiang cattle originated from East Asian taurine ancestry (Fig. 4G-I, and K). Transcriptome analysis of the muscle tissue of Longlin cattle, a type Chinese indicine breed, and Sanjiang cattle will help further identify the functions of genes derived from the taurine ancestor. The results showed that the genes were differentially expressed in the muscle tissues of Longlin cattle and Sanjiang cattle (Fig. 4J and L), further illustrating their contribution to the evolution of Sanjiang cattle.

## Discussion

Autosomal genome-wide analyses show that cattle breeds in hybrid regions of China contain different backgrounds with different levels of genetic contributions across populations, and selection played a role in shaping the taurine × indicine admixture proportion in hybrid cattle (Zhang et al. 2018). In this study, we first highlighted the taurine × indicine admixture characteristics of Sanjiang cattle. We also dated the main taurine × indicine admixture event, which has shaped today’s genome of Sanjiang cattle. In addition, we combined ancestry analysis and selective scanning to determine whether Sanjiang cattle inherited adaptive advantages of ancestral populations under selection pressure. As expected, a series of candidate regions were identified in Sanjiang cattle, including genes related to meat quality from taurine cattle and immune-response- and heat-tolerance-related genes in haplotypes of indicine origins. These genomic fragments contributed to the formation of the Sanjiang cattle genome. However, some individuals contain components of European taurine cattle and Indian indicine cattle, which are inseparable from the blind introduction of local people, which will seriously lead to the loss of local cattle characteristics.

The eastward migration of taurine cattle from the domestic center, West Asia, reached the northern part of China between 5,000 and 4,000 years ago. Three thousand years ago, indicine cattle migrated to China. This has led to the emergence of hybrid breeds. Hybridization of taurine and indicine cattle improves economic traits and adaptation in taurine–indicine transition zones. Our results presented two main types of ancestry of Sanjiang cattle with Chinese indicine (56%) and East Asian taurine (33%) ancestries. The nucleotide diversity of Sanjiang cattle is lower only than that of Chinese indicine, which reflects that the cross between taurine and indicine cattle is the main contributor to the increasing genomic diversity of Sanjiang populations. The analysis of the number of SNPs and LD decay positioned Sanjiang cattle between the *B. taurus* and *B. indicus,* showing generally consistent results of nucleotide diversity. In addition, Sanjiang cattle exhibit larger amounts of short/medium ROH in comparison to other breeds analyzed in this study. We also identified admixture in Sanjiang cattle as a more recent event approximately 30 generations ago, which showed that Sanjiang cattle are a recent breed and indicate that the higher breeding potential of Sanjiang cattle remains to be exploited. Our results now provide a time-scale reference for recent admixture events in native cattle.

Admixture between populations provides an opportunity to study biological adaptation and phenotypic variation. Admixture studies rely on local ancestry inference for hybrid individuals. In our study, we applied LOTER to infer local ancestry combined with selection analysis to obtain the ancestry of selection signatures in Sanjiang cattle. These excessive fragments annotated genes involved in important biological processes, such as immune regulation, stress reaction, and lipid metabolism, which may reflect adaptation to the local environment and artificial breeding during the formation of Sanjiang cattle, providing more accessible genomic information for local ancestral inference about admixture processes.

Sanjiang cattle are an excellent indigenous breed for both labor and meat, which was historically important to local beef production in Sichuan and still exhibits better meat quality today. Among excessive segments inherited from East Asian taurine, some genes associated with lipid metabolism and skeletal muscle development were also under positive selection, such as *SIGMAR1*, *CCL21*, *PPARG*, *ADAMTS9*, and *CNTFR*. *SIGMAR1* is a ubiquitously expressed multifunctional interorganelle signaling chaperone protein that plays a diverse role in cellular survival, including lipid metabolism, which regulates the compartmentalization of ER-synthesized neutral lipids (triglycerides and cholesteryl esters) (Aishwarya et al. 2022; Yang et al. 2020). *CCL21* is considered an adipokine (Namya et al. 2019) associated with fat expansion and metabolic parameters in juvenile rats (Lizarraga-Mollinedo et al. 2022). *PPARG* plays a significant role in lipid metabolism, adipocyte differentiation and fatty acid storage (Argmann et al. 2005; Auwerx 1999). Reportedly, *PPARG* affects not only backfat thickness but also meat quality by affecting fat content and composition in cattle (Fan et al. 2011; Goszczynski et al. 2016; Sevane et al. 2013). In addition, *ADAMTS9* can regulate insulin sensitivity and the levels of mitochondrial complexes in skeletal muscles (Graae et al. 2019), which is a functional molecular marker for improving growth traits in goats (Jungers et al. 2005; Tang et al. 2019). *CNTFR* is expressed in skeletal muscle, with upregulated expression in response to muscle damage and hindlimb unweighting, and negatively regulates fat deposition in rats (Guillet et al. 1998; Kami et al. 2000; Weis et al. 1998). In addition, *CNTFR* is associated with the average daily gain and feed efficiency in beef cattle (Abo-Ismail et al. 2018; Serão et al. 2013). These results implied that the ancestral segments inherited from East Asian taurine cattle could contribute to meat quality traits of Sanjiang cattle.

Compared to commercial northern breeds, indigenous cattle, such as Sanjiang cattle, exhibit genetic advantages in terms of disease resistance, heat tolerance, and adaptation to local environmental conditions. In addition to selection pressures for meat quality traits, Sanjiang cattle had to cope with hot and humid weather, which is why they are assumed to have developed thermotolerance and robustness. Regarding the immune response to infections and reproduction, heat tolerance is one of the main indicators of adaptability to harsh environments. Generally, indicine cattle have stronger adaptability and resistance than taurine cattle to heat, parasites, and infectious diseases (Fernandes Júnior et al. 2020). Indicine cattle found across southern China have been better adapted to local environments. These adaptations would have facilitated indicine introgression into central taurine populations and the dispersion of crossbred animals. Ancestry fragments of Chinese indicine origin may reflect adaptive functions. As in our results, a set of important genes associated with the immune response and stress reaction were putatively positively selected. For example, the *PALB2* tumor suppressor plays key roles in DNA repair and has been implicated in redox homeostasis, thereby promoting antioxidant gene expression (Guo et al. 2015; Xia et al. 2006). Another gene, *DCTN5*, may have immune-related functions in sheep (Habermann et al. 2001; Salavati et al. 2019). Fc receptor–like (FcRL) proteins are an ancient multigene family of transmembrane proteins that share ancestors with classic FcRs and are preferentially expressed in the B-cell lineage (Davis 2007). The *FCRL4* and *FCRL5* genes are involved in immune responses (Cancro and Tomayko 2021; Kim et al. 2019). Long-term exposure to hot and humid environments will increase the animal's respiratory rate and metabolism, affecting the development of the heart and lungs. Among excessive segments inherited from Chinese indicine cattle, three genes related to adaptation to hot and humid environments were annotated. For example, the *ECE2* gene, as a potential candidate autoantigen (Smith-Anttila et al. 2017), is known to act in human brain and heart development, along with other processes crucial to cattle embryonic development (Heather et al. 2011; Yanagisawa et al. 2000). *NDUFAB1*, known as mitochondrial acyl carrier protein, acts as a powerful cardio-protector by conferring greater capacity and efficiency of mitochondrial energy metabolism in response to stressful conditions (Hou et al. 2018). *NOD1* is widely expressed in the heart and lung and is an important mediator of endoplasmic reticulum-induced inflammation in mouse and human cells (Berrington et al. 2010; Keestra-Gounder et al. 2016), acting during heat stress in cattle (Bhanuprakash et al. 2017). As discussed previously, these genes act on immune system activation and hot adaptation in response to environmental stress and are important candidate genes that affect tropical adaptation. Therefore, we concluded that redundant segments from Chinese indicine could contribute to heat and humidity adaptation in Sanjiang cattle.

## Conclusions

By analyzing the whole-genome data of Sanjiang cattle, we generally understood the genetic diversity of Sanjiang cattle and multidimensionally explored the population structure of Sanjiang cattle. In addition, we found the mosaic genome of indigenous Chinese cattle to be a unique genetic resource related to important economic traits and climatic adaptation traits within Sanjiang cattle. Importantly, animal adaptation to the tropics is directly related to the ability to survive and grow in the presence of local environmental stressors. Therefore, our results will provide new information to understand the complex history of breed formation of indigenous Chinese cattle and provide a basis for genetic breeding and resource protection in Sanjiang cattle.

## Methods

### Samples and sequencing

We collected ear tissue samples from 10 Sanjiang cattle (Table S1) and used a standard phenol–chloroform method to extract the genomic DNA (Reid 1991). Paired-end libraries with an average insert size of 350 bp were built for each individual, with an average read length of 150 bp. WGS was performed using Illumina NovaSeq instruments at Novogene Bioinformatics Institute, Beijing, China. To explore possible ancestral components and further understand the genetic diversity of Sanjiang cattle, according to the report of Chen et al. (2018), 70 samples from five continental groups worldwide were added as control groups, including European taurine (Angus and Simmental cattle), East Asian taurine (Hanwoo and Tibetan cattle), Indian indicine (Brahman, Gir, Hariana, Nelore, Sahiwal, and Tharparkar cattle) and Chinese indicine (Wenshan, Wannan, Guangfeng, Ji'an, and Jinjiang cattle) (Table S1). Finally, a total of 80 samples were used in this study.

### Read mapping and SNP calling

BWA-MEM (v0.7.13-r1126) (Li and Durbin 2009) was used to align the clean reads to the *B. taurus* reference assembly ARS-UCD1.2 with default parameters. Picard tools (http://broadinstitute.github.io/picard) were used to identify and filter duplicate reads (REMOVE_DUPLICATES = true). Genome Analysis Toolkit 3.8 (GATK) (Nekrutenko and Taylor 2012) was used to detect SNPs. The “BaseRecalibrator” module of GATK was used for base quality score recalibration. The modules “HaplotypeCaller”, “GenotypeGVCFs” and “SelectVariants” of GATK were used to call the raw SNPs. Moreover, “VariantFiltration” was used to filter the raw SNPs based on the hard filtering parameters “QD < 2.0, FS > 60.0, MQ < 40.0, MQRankSum < -12.5, ReadPosRankSum < -8.0 and SOR > 3.0” and the mean sequencing depth of variants (all individuals) “ < 1/3 × and > 3 × ”. Afterward, a transcript FASTA file for the database was built using the retrieve_seq_from_fasta.pl module of ANNOVAR based on the annotation file (GCF_002263795.1_ARS-UCD1.2_genomic.gff) of the *B. taurus* reference genome. Functional annotation for each SNP was performed using ANNOWAR (Wang et al. 2010).

### Detection of genetic diversity

VCFtools (Danecek et al. 2011) was used to estimate the nucleotide diversity (*θπ*) of each breed, keeping a window size of 50 kb and a step size of 20 kb. PopLDdecay software (Zhang et al. 2019) was used to calculate and visualize the LD decay with physical distance between SNPs, and the same number of individuals were randomly selected for each breed/population using a Python script. ROHs were identified using the “–homozyg” option in PLINK (Purcell et al. 2007). The parameters were as follows: (1) –homozyg-window-snp 100; (2) –homozyg-density 200; (3) –homozyg-window-het 1; (4) –homozyg-kb 100; (5) –homozyg-window-threshold 0.05.

### Population structure and phylogenetic analysis

PLINK (Purcell et al. 2007) was used to prune the SNPs in high levels of pairwise LD with the parameter (–indep-pairwise 50 5 0.2) for PCA and ADMIXTURE analysis (Chen et al. 2020). Relatedness among each individual of Sanjiang cattle was calculated using the kinship coefficient estimator implemented in KING (Manichaikul et al. 2010). We used smartPCA of the EIGENSOFT v5.0 package to estimate the eigenvectors for PCA (Shen et al. 2021). Population structure analysis was performed by ADMIXTURE v1.3 (Alexander & Lange 2011) with the kinship (*K*) parameter set from 2 to 4. For phylogenetic analysis, we used PLINK to calculate the matrix of Hamming distances between pairs of individuals, MEGA v10.0 (Kumar et al. 2018) to construct the NJ tree and iTOL (Letunic & Bork 2019) for visualization.

### Local ancestry inference

Beagle v4.1 (Browning and Browning 2007) was used to conduct haplotype-phase inference and missing allele imputation with default parameters. The time of admixture of Sanjiang cattle was estimated by ALDER (Loh et al. 2013) and fastGLOBETROTTER (Wangkumhang et al. 2022) using the default parameters. LOTER (Dias-Alves et al. 2018) was used to infer taurine and indicine ancestry along the genomes of Sanjiang cattle. We selected Chinese indicine, East Asian taurine, European taurine, and Indian indicine groups as reference panels based on the population structure. Then, the length and frequency of ancestral segments in each reference group were calculated. To detect a high proportion of fragments with an ancestry, the ancestry-specific haplotypes for each fragment were compared to the total number of ancestry-specific haplotypes for all fragments, with regions of significance having a *P* value < 0.01 (*Z* test). The ideogram package (Hao et al. 2020) in R was used to draw chromosome maps to visualize excessive segments of Chinese indicine and East Asian taurine cattle based on the *B. taurus* reference genome. Functional enrichment analysis was performed on the list of genes within the detected excessive segments by KOBAS v3.0 (http://kobas.cbi.pku.edu.cn/) (Bu et al. 2021).

### Selective sweep identification

We detected the selection signatures within Sanjiang cattle by calculating two different statistics, the CLR (Nielsen et al. 2005) and *iHS*. Then, the CLR test was carried out by using SweepFinder2 (DeGiorgio et al. 2016) in nonoverlapping 50 kb windows. Genotype files were phased and imputed using Beagle (Browning & Browning 2007), and* iHS* was calculated using Selscan v2.0 (Szpiech and Hernandez 2014) with the same window size used for the CLR. We calculated the empirical *P* values for the CLR and iHS windows. The windows whose empirical *P* values were in the top 1% of values for both methods were considered candidate regions of selection.

In addition, the fixation index (*F*_ST_) and large differences in genetic diversity (*θπ*- ratio) were calculated to identify the potential selection regions between Sanjiang cattle and the reference groups, Hanwoo cattle and Chinese indicine cattle. We estimated the genome-wide distribution of* F*_ST_ values using VCFtools (Danecek et al. 2011) in 50 kb windows with a 20 kb step size to investigate pairwise genetic differentiation. The *θπ*-ratio was calculated as ln(π Sanjiang) − ln(π Reference), where πSanjiang and πReference are the nucleotide diversity values for the Sanjiang cattle and reference groups, respectively. The *θπ*-ratio was calculated in the same parameters as *F*_ST_. Significant genomic regions were identified by a *P* value < 0.01. The genomic regions identified by at least two methods were considered candidates for positive selection. Tajima’s *D* was calculated through VCFtools for each candidate gene. KEGG pathways and GO terms were analyzed using KOBAS v3.0 (http://kobas.cbi.pku.edu.cn/) (Bu et al. 2021) to better understand the gene functions. When the corrected *P* value was less than 0.05, the results were considered significantly enriched.

### RNA-Seq and differentially expressed gene analysis

To further confirm the candidate genes under positive selection in Sanjiang cattle, we downloaded transcriptomic data of lung and muscle in adult Sanjiang cattle from NCBI (PRJNA512958) and extracted total RNA from lung of three Longlin cattle (Chinese indicine cattle) for sequencing using Illumina HiSeq X Ten system to generate 150 bp paired-end reads. We used HISAT2.1.0 (Kim et al. 2015) and StringTie (Pertea et al. 2015)  software to map and assemble the reads based on the taurine reference genome assembly (ARS-UCD1.2). The differentially expressed genes (DEGs) of the lung were compared in transcripts per kilobase million (TPM) between Sanjiang and Japanese black cattle (East Asian taurine cattle). Data for Japanese black cattle were obtained from the Wagyu Genome Database (WGDB; https://wagyu.hgc.jp/open/download/rnaseq/). In addition, a Python script was utilized to convert the StringTie result into DEseq2 (Love et al. 2014). Finally, DEGs of muscle between Sanjiang and Longlin cattle were analyzed by the DEseq2 package in R. The adjusted *P* value < 0.01 and |log_2_(Fold Change)|> 1 were used as the cutoff value to determine the DEGs.

## Supplementary Information

**Additional file 1. Fig S1.** The distribution map of the Sanjiang cattle and neighboring Sanjiang cattle breeds. **Fig S2.** The genome-wide distribution of nucleotide diversity in each breed is presented in 50 kb windows with 20 kb steps.**Fig S3.** Number of SNPs identified in each breed with respect to the reference genome. High and low bars represent the numbers of all SNPs (lefty-axis) and breed-specific SNPs (right y-axis), respectively. **Fig S4.** Genome-wide average LD decay estimated for each breed. **Fig S5.** Runs of homozygosity (ROHs) patterns of all individuals from each cattle geographic groups. 

**Additional file 2. Table S1.** Summary of sequencing data. **Table S2.** Distribution of SNPs in different regions of the genome annotated by ANNOVAR. **Table S3.** Relatedness among each individual of Sanjiang cattle was calculated using KING. **Table S4.** Runs of homozygosity (ROHs) patterns of all individuals from each cattle geographic groups. **Table S5.** The gene annotation results of high-frequency segments (frequency≥0.75, length≥1000bp) detected by LOTER software. **Table S6.** KEGG and GO results from the enrichment analysis of genes with excessive Chinese indicine proportions. **Table S7.** KEGG and GO results from the enrichment analysis of genes with excessive East Asian taurine proportions. **Table S8.** A summary of genes from CLR in Sanjiang cattle. **Table S9.** A summary of genes from iHS in Sanjiang cattle. **Table S10.** A summary of genes from *F*_ST_ between Sanjiang cattle (target population) and Hanwoo (reference population). **Table S11.** A summary of genes from *θπ*-ratio between Sanjiang cattle (target population) and Hanwoo (reference population). **Table S12.** KEGG pathway analysis of candidate genes from Sanjiang cattle (target population) and East Asian taurine (reference population) comparison. **Table S13.** Twenty-seven missense SNPs with high frequency alleles present in Sanjiang cattle (>70%) with an excess of indicine ancestry, but low frequency of the taurine (MAF < 0.2). **Table S14.** A summary of genes from *F*_ST_ between Sanjiang cattle (target population) and Chinese indicine (reference population). **Table S15.** A summary of genes from *θπ*-ratio between Sanjiang cattle (target population) and Chinese indicine (reference population). **Table S16.** KEGG pathway analysis of candidate genes from Sanjiang cattle (target population) and Chinese indicine (reference population) comparison. 

## Data Availability

Sequences are available from GenBank with the Bioproject accession number PRJNA668518.

## Abbreviations

SNP: Single nucleotide polymorphism
WGS: Whole-genome sequencing
*θ*π: Nucleotide diversity
*θ*π-ratio: Nucleotide diversity ratio
CLR: Composite likelihood ratio
*F*_ST_: Fixation index
GO: Gene Ontology
KEGG: Kyoto Encyclopedia of Genes and Genomes
NJ: Neighbor-joining
PCA: Principal component analysis
ROH: Run of homozygosity
LD: Linkage disequilibrium
MAF: Minor allele frequency
DEGs: Differentially expressed genes
TPM: Transcripts per million
