# Supplementary material for: Local ancestry and selection in admixed Sanjiang cattle
Source: Stress Biol. 2023 Aug 3;3(1):30. doi: 10.1007/s44154-023-00101-5 (PMC10441984; doi:10.1007/s44154-023-00101-5)
Supplement: Supplementary file 1 — Additional file 1. Fig S1. The distribution map of the Sanjiang cattle and neighboring Sanjiang cattle breeds. Fig S2. The genome-wide distribution of nucleotide diversity in each breed is presented in 50 kb windows with 20 kb steps.Fig S3. Number of SNPs identified in each breed with respect to the reference genome. High and low bars represent the numbers of all SNPs (lefty-axis) and breed-specific SNPs (right y-axis), respectively. Fig S4. Genome-wide average LD decay estimated for each breed. Fig S5. Runs of homozygosity (ROHs) patterns of all individuals from each cattle geographic groups. [file 44154_2023_101_MOESM1_ESM.docx]

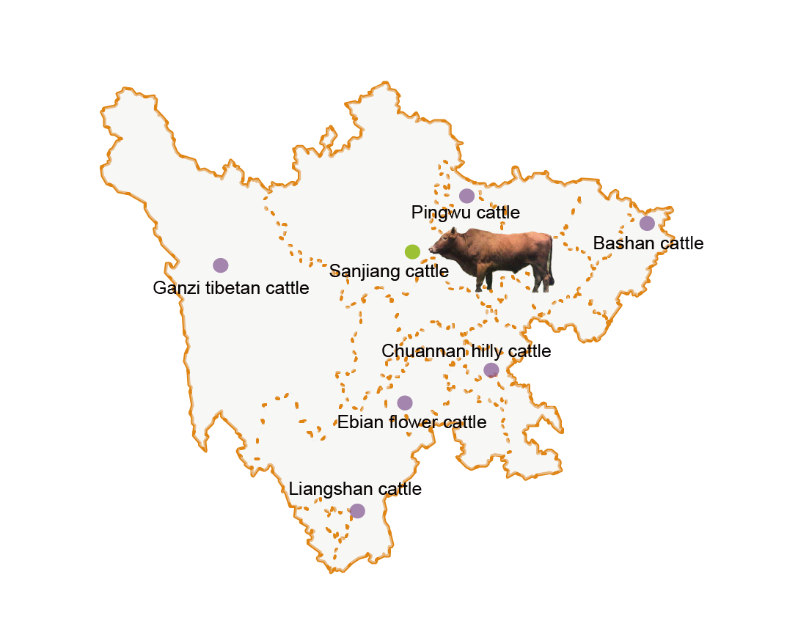


Fig S1. The distribution map of the Sanjiang cattle and neighboring Sanjiang cattle breeds


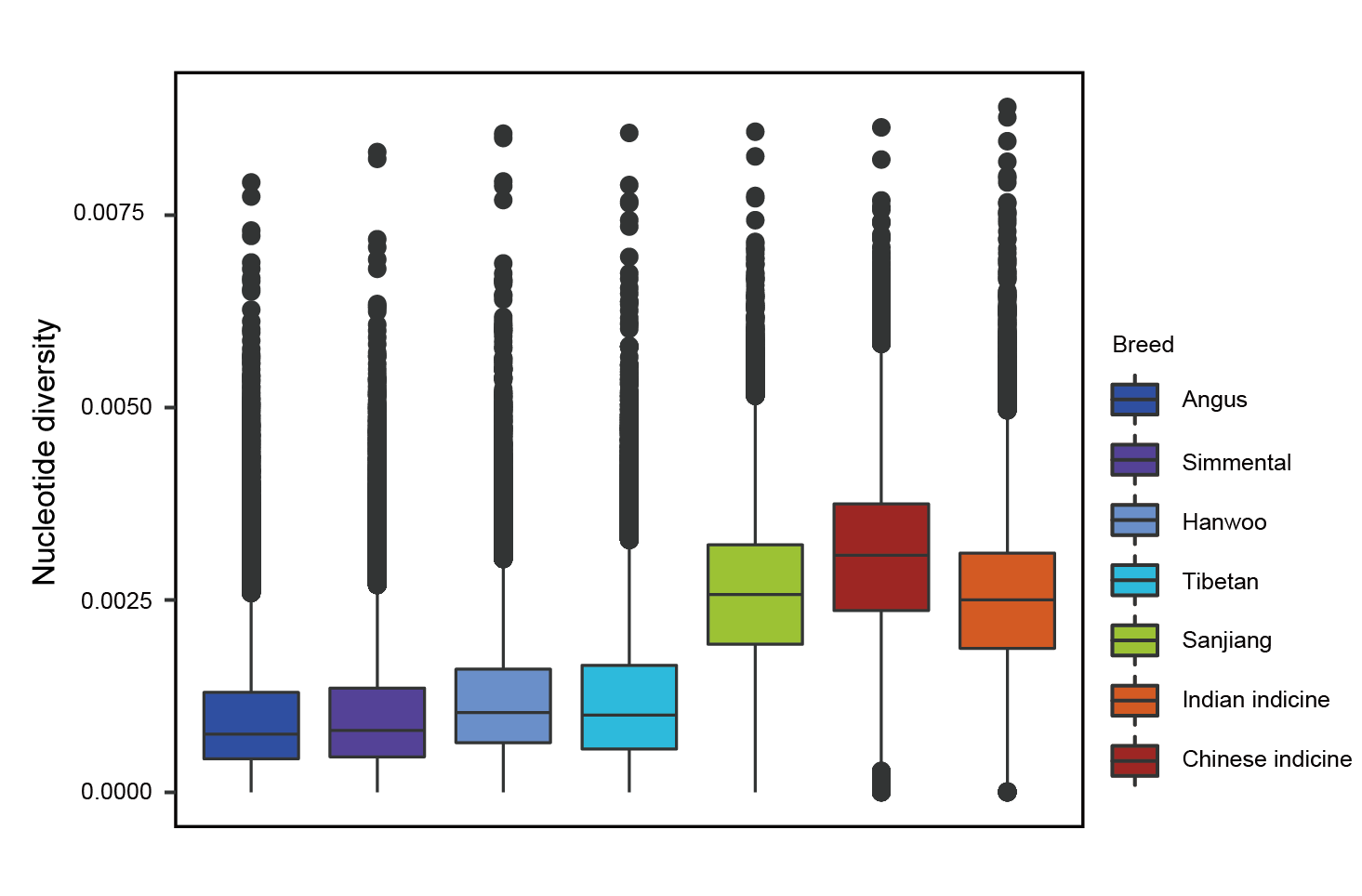


Fig S2. The genome-wide distribution of nucleotide diversity in each breed is presented in 50 kb windows with 20 kb steps.


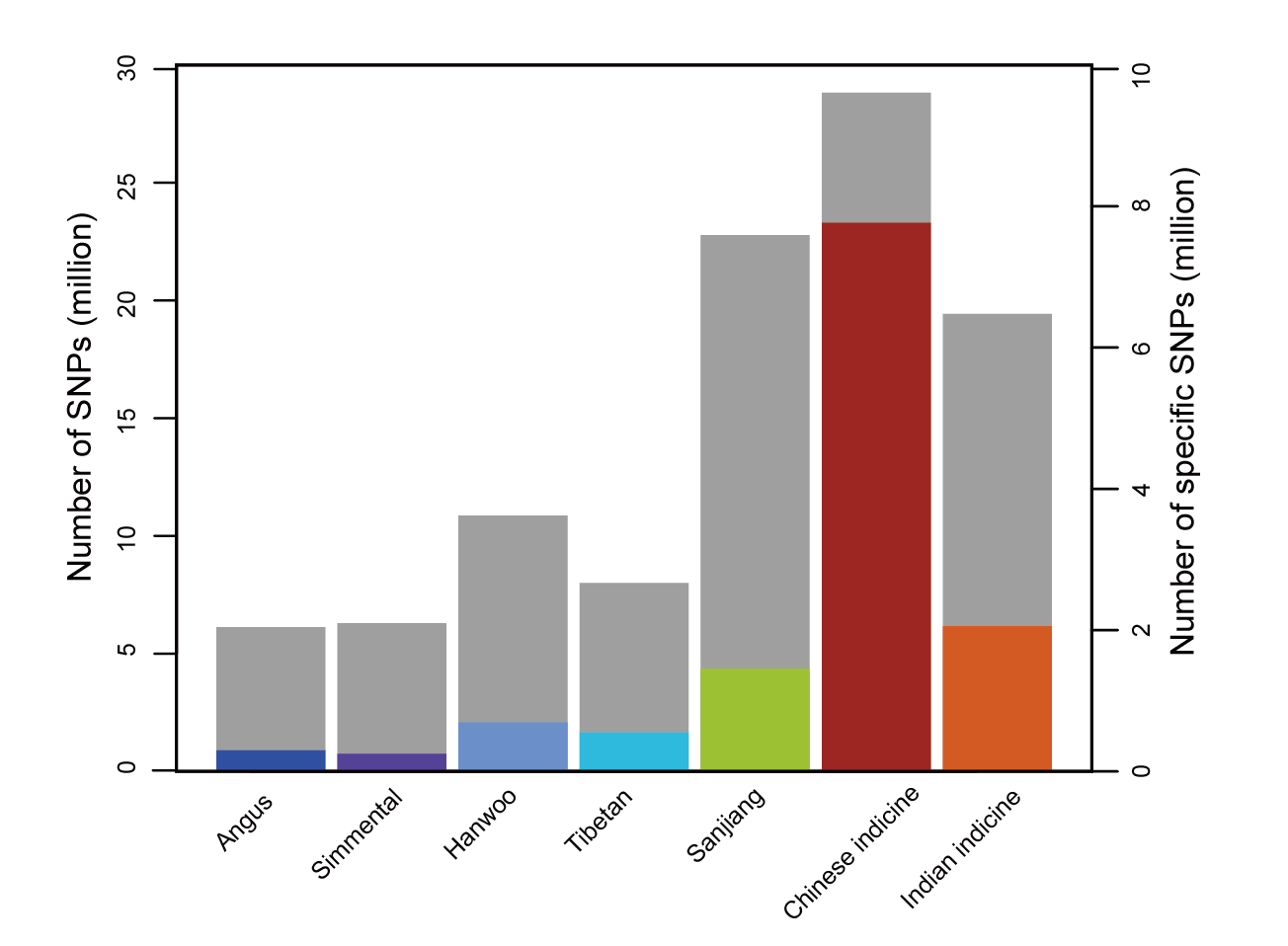


Fig S3. Number of SNPs identified in each breed with respect to the reference genome.

High and low bars represent the numbers of all SNPs (left y-axis) and breed-specific SNPs (right y-axis), respectively.


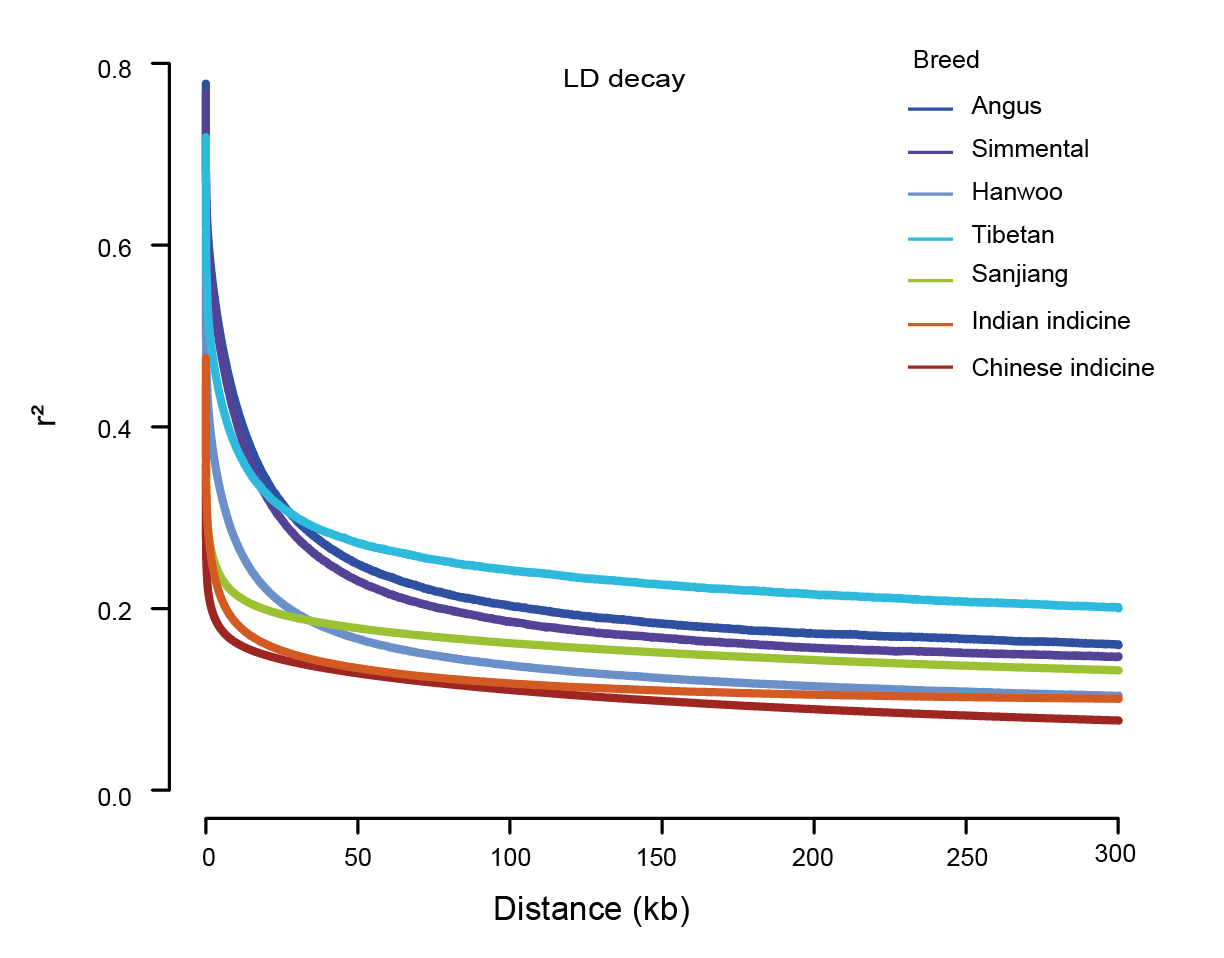


Fig S4. Genome-wide average LD decay estimated for each breed.


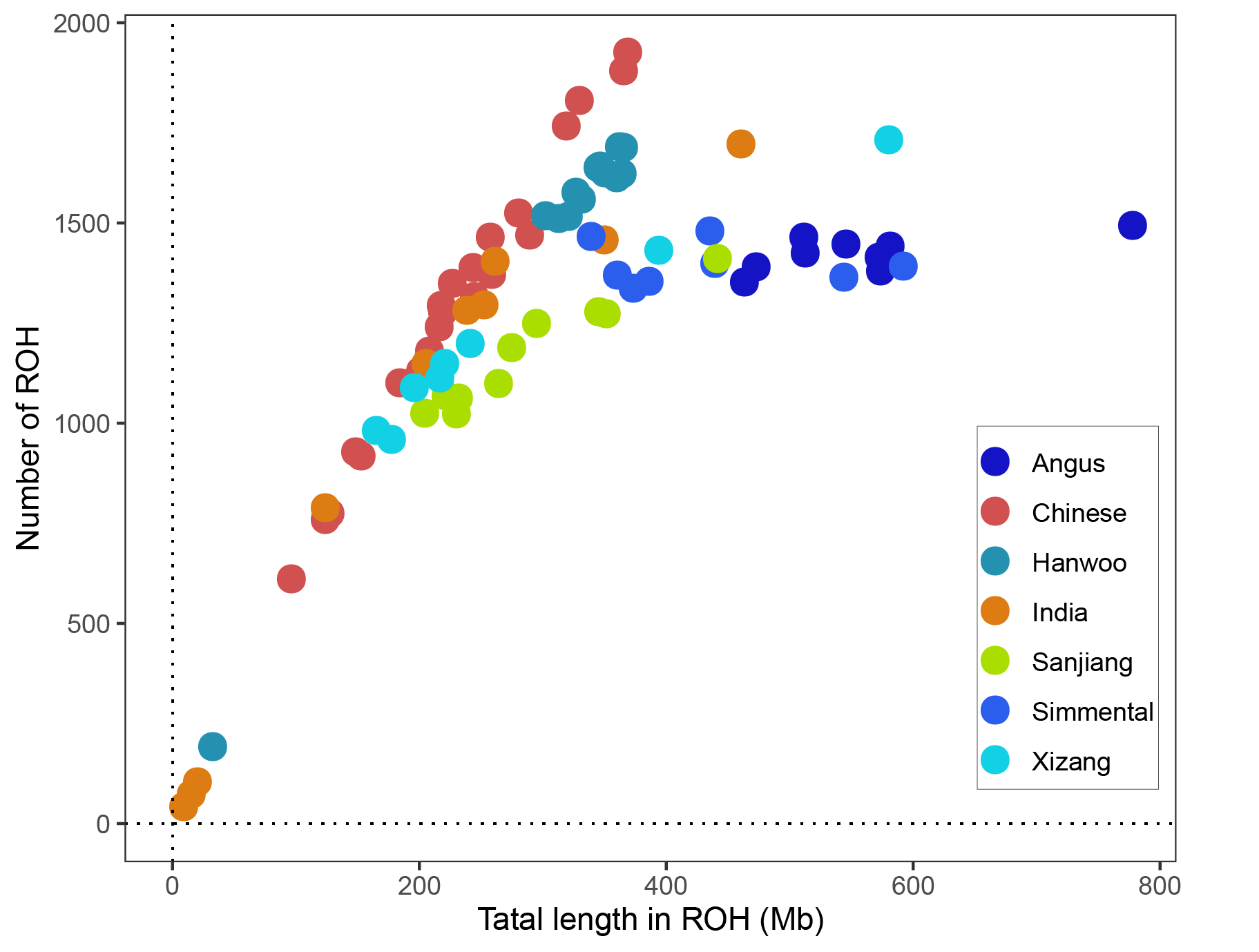


Fig S5. Runs of homozygosity (ROHs) patterns of all individuals from each cattle geographic groups.
